# Platelet-Rich Plasma in Diabetic Foot Ulcer Healing: Contemplating the Facts

**DOI:** 10.3390/ijms252312864

**Published:** 2024-11-29

**Authors:** Jacob Smith, Vikrant Rai

**Affiliations:** Department of Translational Research, Western University of Health Sciences, Pomona, CA 91766, USA; jacobsmith@westernu.edu

**Keywords:** diabetic foot ulcers, nonhealing, platelet-rich plasma, constituents, mode of administration, enhanced healing, epigenetics

## Abstract

Diabetic foot ulcers (DFUs), debilitating complication of diabetes, often lead to amputation even in the presence of current advanced treatment for DFUs. Platelet-rich plasma (PRP) containing growth factors and other proteins has been suggested as a potent therapeutic in promoting DFU healing. PRP is safe and effective in improving the DFU healing rate, decreasing healing time, and making chronic wounds viable for treatment. Though PRP is safe and effective in promoting DFU healing, there are inconsistencies in clinical outcomes. These varying results may be due to various concentrations of PRP being used. Most studies report dosage and timing, but none have reported the concentration of various factors. This is important, as the concentration of factors in PRP can vary significantly with each preparation and may directly impact the healing outcome. This critical review discusses the limiting factors and issues related to PRP therapy and future directives. A systematic search of PubMed and Google Scholar was performed with keywords including diabetic foot ulcer, ulcer healing, platelet-rich plasma, DFU treatment, and PRP limitations and efficacy, alone or in combination, to search the related articles. The articles describing DFU and the use of PRP in DFU healing were included. The existing literature suggests that PRP is effective and safe for promoting DFU healing, but larger clinical trials are needed to improve clinical outcomes. There is a need to consider multiple factors including the role of epigenetics, lifestyle modification, and the percentage composition of each constituent in PRP.

## 1. Introduction

Diabetes mellitus is a chronic condition where the body cannot produce sufficient levels of insulin (type 1) or fails to effectively utilize the insulin it does produce (type 2) [1]. Lack of insulin leads to hyperglycemia (high levels of blood glucose), the clinical indicator for diabetes [1]. Each year, 1.5 million deaths are attributed directly to diabetes, with an estimated 422 million people suffering from the disease worldwide [2]. One major source of preventable morbidity in adults with diabetes is diabetic foot ulcers (DFUs), which can contribute to various outcomes such as infection, hospitalization, amputation, and death [3]. Over the past several years, the amputation incidence has been reported to have increased by as much as 50% in some areas [3]. DFUs also present a heavy financial burden on patients; the mean cost per patient per year was USD 3368 for ulceration only, while amputations ranged from USD 10,468 for minor amputation and USD 30,131 for major amputation in 2021 [4]. DFUs are most common in patients with type 2 diabetes, longer duration of diabetes, and the elderly. They are most prevalent in North America [5].

Nonhealing or chronicity of DFUs is due to the presence of chronic inflammation, decreased angiogenesis, altered extracellular matrix (ECM) formation, and prolongation of the inflammatory phase of wound healing [6,7]. Treatment for DFUs consists of conventional wound treatment methods (such as infection management, wound discharge, and wound dressing), as well as blood glucose control [8]. These treatments have proven to be inadequate, with approximately 23% of diabetic foot ulcers not healing within one year and recurrence within one year of healing occurring among roughly 40%. Along with the current treatment, topical oxygen or hyperbaric oxygen therapy [9,10], stem cell therapy [11], negative pressure wound therapy [12], exosome therapy [13], and platelet-rich plasma therapy to promote angiogenesis [11,14] and ECM formation have been discussed in the literature. However, none has shown statistically significant clinical outcomes for DFU healing. Growth factors have been suggested to improve wound healing in DFUs [15]; however, the only Food and Drug Administration (FDA)-approved therapy to promote DFU healing is platelet-derived growth factor-BB (PDGF-BB) therapy [16]. Fibroblasts, vascular smooth muscle cells, endothelial cells, pericytes, keratinocytes, and interaction between them, as well as with immune cells, play a critical role in wound healing and growth factors regulate the proliferation, migration, and differentiation of these cells during wound healing [17].

Platelet-rich plasma (PRP), a mixture of growth factors, cytokines, and interleukins, promotes tissue regeneration via enhanced angiogenesis and ECM modulation [18]. PRP promotes the migration of fibroblasts, epithelial cells, endothelial cells (ECs), and mesenchymal stem cells to promote wound healing [19]. Research studies included in a meta-analysis by OuYang et al. [8] suggest that PRP is effective in improving the DFU healing rate and decreasing the healing time. However, there was no significant difference in ulcer area reduction. Other studies have shown PRP to be an effective and safe supplementary therapy to promote complete healing in DFU [18,20]. PRP therapy to promote DFU healing is still considered adjuvant therapy needing further clinical trials. The role of PRP as an adjuvant therapy may be due to multiple factors, including patient body mass index, smoking status, duration of diabetes, renal status [21], and proportion of various factors in PRP [22]. This review aims to discuss the various aspects of PRP therapy in DFU healing, especially focusing on the factors affecting the efficacy of PRP in DFU healing, followed by the importance of documenting the percentage of each constituent of PRP and its effects on wound healing.

## 2. Materials and Methods

A systematic search of PubMed and Google Scholar was performed with keywords including diabetic foot ulcer, ulcer healing, platelet-rich plasma, DFU treatment, and PRP limitations and efficacy, alone or in combination, to search the related articles. Article titles and abstracts were screened. The full-text review determined the suitability of the articles to include in this review. All duplicate articles and articles in a language other than English were excluded.

## 3. Platelet-Rich Plasma Preparation

**Figure 1:**
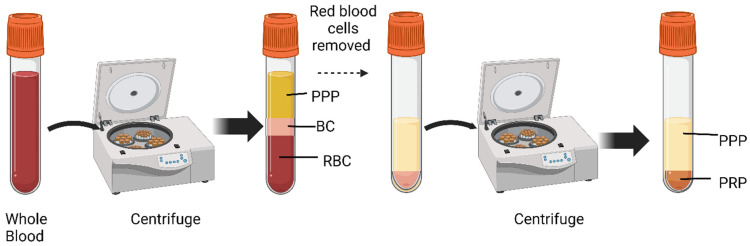
Preparation of platelet-rich plasma. The first centrifugation separates the whole blood into three layers: 1. red blood cell layer (RBC), 2. buffy coat (BC) containing white blood cells and most of the platelets, and 3. platelet-poor plasma (PPP) layer. The second centrifugation of BC and PPP separates all platelets in platelet-rich plasma (PRP) layer and PPP.

Platelet-rich plasma (PRP) is a promising treatment method for diabetic ulcers. Platelets store a variety of growth factors necessary for tissue regeneration and repair [5]. Platelets also have antibacterial properties, inhibiting bacterial growth in the wound [23]. PRP has a higher concentration of platelets than whole blood, with roughly three to five times more platelets than baseline plasma. PRP is traditionally prepared in a two-step process (Figure 1). The blood is first separated into its three components through centrifugation. This results in the formation of three layers: red blood cell layer, white blood cell and platelets layer (referred to as the buffy coat) [24], and a layer of platelet plasma. A second centrifugation is then performed, which further concentrates the platelets into a small volume of plasma known as PRP [24].

## 4. Platelet-Rich Plasma and Wound Healing

Autologous blood refers to the collection and reinfusion of the patient’s own blood or blood components. Allogenic refers to blood transfusions involving collecting and infusing blood/blood components of a compatible donor [25]. PRP can be harvested from both autologous (au-PRP) and allogenic blood (al-PRP) [26]. Au-PRP has previously been applied successfully in chronic refractory wounds, and evidence suggests its efficacy in improving the healing of chronic wounds in DFU [27,28]. Autologous PRP has been documented to be safe and effective in skin wound repair; however, allogenic PRP has potential immunogenicity issues. There are advantages of al-PRP: it is ready to use, and there ia no need to take blood from the patient and process it. Not withdrawing blood from patients prevents the chances of fluid depletion and thrombocytopenia in patients already facing fluid depletion, especially burn patients. Akbarzadeh et al. reviewed ten studies (eight human patient studies and two animal studies) and concluded that al-PRP may be a safe and effective alternative when au-PRP is not available. However, large clinical trials are warranted because of the presence of a lack of a control group, inconsistencies in PRP preparation methods, and treatment regimens among various studies [29].

Complications and diseases coexisting with DFU, such as anemia, thrombosis, malnutrition, and more, can prevent the utilization of au-PRP in some patients [26]. In the presence of diabetes, PRP efficacy may be hindered due to poor platelet numbers and reduced cell activity [26]. These limitations may be surmounted by utilizing allogenic PRP obtained from a healthy individual. PRP can be effectively administered in a variety of ways. One such way is through autologous gels. Autologous PRP gels have been shown to accelerate wound healing in DFU, compared to traditional antiseptic ointment dressing. The use of platelet gels showed a lower rate of wound infections, indicating they might have an antimicrobial effect [30]. PRP consists of cytokines, fibrin scaffolds, and growth factors that stimulate cellular differentiation, cell proliferation, and extracellular protein synthesis [21]. It is a minimally invasive and safe method to promote wound healing [28].

**Figure 2:**
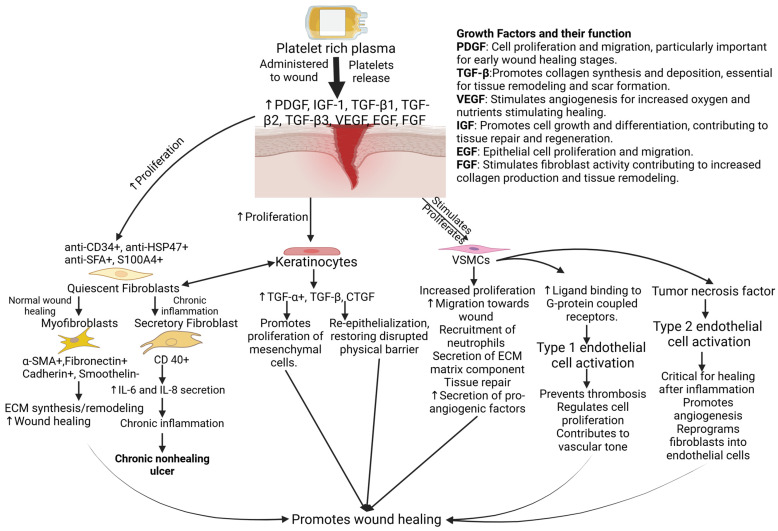
Molecular and cellular role supplemented by platelet-rich plasma (PRP) when administered to wound. The growth factors stored in platelets are secreted and stimulate the proliferation and migration of fibroblasts, keratinocytes, vascular smooth muscle cells (VSMCs), and endothelial cells toward the wound area. These cells and their interaction in-between and with immune cells help in promoting wound healing by suppressing inflammation and promoting tissue regeneration. PDGF—platelet-derived growth factor, EGF—epidermal growth factor, FGF—fibroblast growth factor, VEGF—vascular endothelial growth factor, IGF—insulin-like growth factor, TGF-β—transforming growth factor-beta, CD34—cluster of differentiation 34, HSP47—heat shock protein 47, SFA—fibroblast-specific surface antigen, SMA—smooth muscle actin, ECM—extracellular matrix, and IL-interleukin.

In wound healing, PRP releases a variety of bioactive molecules that have been stored in the platelets, such as growth factors, lysosomes, and cytokines [31]. There is a multitude of growth factors present in PRP, such as platelet-derived growth factor (PDGF), insulin-like growth factor (IGF-1), transforming growth factors (TGF) β1, β2, and β3, vascular endothelial growth factor (VEGF), epidermal growth factor (EGF), and various others [32]. The role of these factors in the differentiation, proliferation, and migration of various cells in wound healing has been discussed [17] (Figure 2).

This suggests that varying concentrations of growth factors and other components in PRP may affect wound healing. Research has shown that growth factor concentration and catabolic cytokine concentrations are influenced by the cellular composition of the PRP. Anabolic signaling is increased by platelets, while catabolic signaling is increased by leukocytes. Thus, evaluating the content of PRP is very important, because the biological effects are influenced by the PRP content [33]. PRP injectate is typically autologous and differs based on protocol and specific manufacturer [34]. The optimal platelet concentration for PRP has not yet been identified, but PRP has been defined as having a minimum platelet concentration of 1,000,000 platelets/µL [35]. PRP regulates inflammation by reducing the production of inflammatory cytokines and promotes angiogenesis and re-epithelialization [36]. PRP promotes wound healing by metabolic reprogramming in fibroblasts via stimulating glycolytic enzyme activity and regulating energy metabolism, as well as by increasing antioxidant production to prevent reactive oxygen species formation [37]. PRP influences the molecular mechanisms and biological processes essential for wound healing. Given that the concentrations of its components can vary due to different preparation methods and batch variations, these fluctuations may impact the effectiveness of PRP in healing diabetic foot ulcers. In the following section, we have described the factors affecting PRP efficacy in wound healing.

## 5. Factors in PRP Affecting DFU Healing

PRP as a treatment for DFU has been shown to have mixed results. There are multiple reasons for the varying efficacy of PRP in promoting DFU healing. Studies have shown that various platelet concentrates have distinctly different release kinetics [38]. Too low of a platelet concentration would not be effective in promoting wound healing, while too high could prove destructive to the process of wound healing [34]. Product preparation of PRP makes a significant difference to the platelet composition of PRP and, in turn, affects its efficacy. A study on intestinal healing of rats demonstrated that PRP concentration plays a critical role in PRP efficacy. PRP may have positive effects on wound healing up to a certain level, but negative effects begin to occur when the dosage is too high [34]. Concentration of PRP refers to the total amount or percentage of various factors, platelets, and proteins present, while dosage refers to how much is administered to the patient. Dosage is dependent on the concentration; the higher the concentration of PRP, the lower the dosage that is needed. The dose of PRP to be administered also depends on the concentration of various factors and their role in molecular mechanisms involved in healing. Considering PRP concentration and dose is important because it affects clinical outcomes [39]. Dosages of 50,000 to 100,000 have been shown to have a positive impact on proliferation, while doses of 500,000 have been shown to induce cell death [34,40]. Studies indicate that it may be possible to obtain a standard platelet concentration through the adjustment of the centrifugal force of each sample individually [41].

The proportion of numerous factors within PRP plays a critical role in PRP effectiveness. Meta-analyses have shown that administration of various growth factors improves DFU significantly compared to the control [22]. One such growth factor is EGF, which has been shown to have the potential of healing in chronic DFUs, as compared to control [42]. EGF is needed for the regeneration of skin because it promotes epithelial cell growth and differentiation [43]. Topical application of recombinant human EGF (rhEGF) may improve foot ulcerations significantly, while more severe ulcers may require rhEGF injections [42]. EGF concentration within PRP could significantly alter the effectiveness of PRP. VEGF is involved in the stimulation of new blood vessels (angiogenesis), which is necessary to deliver nutrients and oxygen to the healing wound. VEGF concentrations in PRP may play a role in PRP efficacy. Stem cells from PRP with stromal vascular fraction increase growth factor concentration, including the concentration of VEGF, and promote wound healing [44]. Another crucial factor in the viability of wound healing is TGF-β, which is involved in inflammation regulation, cell differentiation, angiogenesis, re-epithelialization, granulation tissue formation, and extracellular protein synthesis. TGF-β is also crucial for wound healing, as it stimulates fibroblast differentiation into myofibroblasts [43,45]. This suggests that growth factors play a critical role during wound healing by monitoring cell differentiation and proliferation, extracellular matrix remodeling, and wound maturation [17]. There is currently a discrepancy in the effectiveness of PRP, with some studies citing 95% effectiveness [46,47,48], while others cite roughly 63.7% [49]. These differences are likely due to individual platelet concentrations within the PRP. Gender and age have not been shown to significantly impact the success rates of PRP [49].

Every batch of au-PRP is currently composed of different concentrations of growth factors. PRP derives its ability to stimulate tissue regeneration and repair through growth factors, such as PDGF, EGF, and VEGF. PRP provides many of the growth factors required for wound healing. Platelets present in PRP promote the secretion of these growth factors (PDGP, TGF-β, EGF), resulting in an environment that stimulates wound healing [49]. It is important to know the composition and percentage of each factor, because TGF-β is associated with tissue fibrosis [50] and has its translational challenges. Low levels of EGF are associated with impaired healing, and healing response with EGF is concentration-dependent because of the different fates of EGFR through different internalization pathways with different concentrations of EGF [51]. Thus, it is important to know the concentration of various growth factors in PRP (VEGF, EGF, TGF-β, PDGF, cytokines, fibrin, and other proteins) and to standardize the PRP in effectively treating DFU. Concentrations of various growth factors and other proteins within PRP are not currently documented by most of the published literature (Table 1), leading to inconsistent efficacy results. It is also worth noting that many studies have not mentioned whether they used autologous or allogenic PRP, and some studies used PRP in combination with other factors as a source of stem cells (Table 1).

Various studies using PRP to promote wound healing in DFU suggest that PRP is effective in promoting DFU healing. Gupta et al. treated 60 DFU patients (30 with PRP and 30 with normal saline) and reported an 85.98% reduction in wound area with PRP compared to 81.72% with normal saline after 6 weeks [52]. Rainys et al. reported that among sixty-nine DFU patients (thirty-five treated with PRP and thirty-four in the control group), 25.61% of the treatment group (nine patients) had complete re-epithelization of ulcers, as compared to 17.64% (six patients) in the control group. The remaining patients did not show wound healing [55]. Malekpour et al. involving 90 clean DFU patients (43 treated with PRP gel twice weekly for three weeks) reported an average healing time of 55 days with PRP and 80 days in the control group [56]. Orban et al. [58], with 72 patients (36 in PRP treated group and 36 in the control group), reported wound healing in 86.11% (31/36) in the PRP group and 63.89% (23/36) in the control group with conventional dressing. Gowsick et al., with 174 chronic nonhealing DFU patients (87 treated with PRP and 87 in the control group), reported 65.1% (54/87) healing in the PRP group and 42.7% (35/87) healing in the control group after 12 weeks [64]. Hossam et al. [59], with a total of 80 DFU patients (40 receiving PRP injections and 40 receiving standard care with moist dressings), reported complete wound healing in 95% (38 patients), with 77.8% (28 patients) healing in PRP after 6 weeks and control group after 9 weeks, respectively. These clinical studies taken together suggest that out of 315 patients, 158 were treated with PRP, while 157 were given traditional wound dressings. Of the treatment group, 94 patients obtained total wound healing, while 63 did not. Of the control patients, 64 achieved total wound healing, while 93 did not. This resulted in complete healing of 59% of the treated group and only 40% of the control. These results suggest the efficacy of PRP, but the results are not the same in all studies. Thus, it is important to know the source of PRP, because an earlier study reported that bovine thrombin used in PRP preparation did not inhibit Factor V, but au-PRP might have healed the wound [66]. Another important factor was that the control group in each study was different compared to PRP therapy (gel or dressing or injection). Further, the time for follow-up for wound healing was also different among studies (Table 1). An important unanswered question is the role of genetics and epigenetics in DFU healing [67,68,69] while using PRP, which has not been discussed or evaluated in any study.

Platelet-rich plasma (PRP), platelet leukocyte gel (PLG), platelet-rich fibrin (PRF), plasma rich in growth factors (PRGF), and platelet concentrate (CP) are important autologous biomaterials in regenerative medicine [70]. Autologous platelet leukocyte gel (PLG), or autologous platelet-rich gel (APG), has been shown to significantly improve the healing of DFU, shortening healing time and lowering the amputation rate [71,72]. Advanced platelet-rich fibrin (a-PRF), in combination with hyaluronic acid, may have a complimentary effect on the healing of DFUs [73]. Hyaluronic acid (HA), which has a high molecular weight, has been shown to stimulate anti-inflammatory responses from macrophages [74]. Administration of A-PRF + HA has been demonstrated to increase endothelial cell proliferation, migration, and tube formation, leading to increased granular tissue formation, thus promoting the healing of DFUs [73]. Autologous plasma rich in growth factors (PRGF) membrane is a secure and efficacious treatment for chronic non-healing DFUs [75]. Further, platelet concentrate has also been shown to be effective in improving DFU healing [76]. These results suggest that PRP, PLG, PRF, PRGF, and CP improve DFU healing, and the common denominators are growth factors in these concentrates. PRP is a mixture of concentrated platelets, cytokines, and growth factors and has anti-inflammatory properties; the proportion of each component may affect wound healing.

In addition to the concentrations of each component, treatment method (the way of administering PRP, locally or injected), poor patient selection, contamination of PRP with microbes during blood collection, manufacturing and administration processes, removal of the PRP gel during surgical debridement of the ulcer, patient-related factors including a higher BMI, prolonged diabetes, smoking, renal issues with lower glomerular filtration rate, a very large wound, presence of scar tissue in the wound, infections, and nutritional deficiency in patient may lead to PRP failure or decreased efficacy [21,77,78,79].

## 6. Future Direction: Improving DFU Healing Using PRP

As discussed above, PRP is an effective strategy to promote DFU healing; however, most of the studies suggest the need for larger clinical trials for evidence-based medicine [80]. Conventional treatment for diabetic foot ulcers includes infection management, debridement, sterile dressings, and glucose control [6]. Compared to traditional treatments, patients treated with PRP had increased wound healing rates and shorter healing times [81]. Many studies show that PRP significantly increases wound healing, though they do not focus on predictive factors for PRP success. It has been demonstrated that a longer duration of diabetes, higher body mass index (BMI), and smoking status may all play a role in PRP failure in DFU [21]. This indicates the need to improve methods of using PRP for DFU healing response. Solovieva et al. [82] reported that au-PRP from type II diabetes mellitus (TIIDM) has toxic effects on mesenchymal stem cells, and immobilization of TIIDM on polycaprolactone (PCL) nanofibers obviates the cytotoxic effects on mesenchymal stem cells, along with improving endothelial cell proliferation and adhesion. These findings suggest that immobilization of PRP may be beneficial in promoting angiogenesis and thereby DFU healing. This hypothesis is further supported by the findings that PRP immobilization of PCL nanofibers will increase the time of PRP covering the wound. Manakhov et al. [83] reported that immobilized PRP will cover nearly 50% of the surface after 8 days (long-term stability), and the release of PRP biomolecules depends on pH during the first hours of immersion. Another strategy may be using PRP-derived exosomes. PRP-Exos miR-26b-5p has been found to be effective in improving diabetic wound healing by inhibiting neutrophil extracellular traps (NETs) by targeting MMP-8 [84]. Additionally, improving the technique of PRP preparation, improving patients’ nutritional status, strictly regulating blood sugar, regular exercise, avoiding smoking and alcoholic drinks, and non-steroidal anti-inflammatory drugs and wound care may improve PRP effectiveness [85,86].

## 7. Conclusions

In conclusion, the studies so far using PRP in DFU healing have suggested its efficacy and safety but have proposed the need for larger clinical trials. In addition to knowing the percentage of each component of PRP, the role of epigenetics and genetics should be considered for better outcomes. Further, lifestyle modification, including cardiovascular exercise to improve blood supply; avoiding caffeine, alcohol, and smoking; decreasing weight; blood sugar control; perilesional injection of PRP; and multidisciplinary care including surgeons, podiatrists, endocrinologists, and physical therapists should be considered. Additionally, an increased intake of dark green leafy vegetables, vitamin B, and iron and a decreased intake of food that decreases platelet counts (such as quinine in tonic water, cranberry juice, walnuts, and sesame) may help in improving PRP efficacy.

## Figures and Tables

**Table 1:** Studies conducted using platelet-rich plasma (PRP) for healing response in diabetic foot ulcers (DFUs).

Aim of the Study	Type of Study/Number of Patients	Study Outcome	PRP Constituents’ Concentrations
To evaluate the efficacy of local application of PRP in DFU healing [52].	Randomized control study. 60 noninfected DFUs (30 patients with PRP and 30 with normal saline).	PRP and saline dressing have similar effects on DFU healing, and PRP is no more efficacious.	Not mentioned in the study
To evaluate the efficacy of au-PRP in DFU healing [53].	Prospective study. 150 DFU patients.	PRP promotes wound healing, and the wound did not reopen after 8 months of healing.	Not mentioned in the study
To evaluate the effects of stem cells present in fat (using fat graft) and PRP in DFU healing [54].	Randomized control trial. 18 DFU patients.	Increased neovascularization and graft survival in DFUs.	Not mentioned in the study
To evaluate the effectiveness of au-PRP gel in the treatment of hard-to-heal leg ulcers [55].	Prospective randomized control trial. 69 patients (35 in PRP and 34 in the conventional treatment group).	Au-PRP gel is effective in reducing wound size and promoting granulation tissue formation, but gel application is associated with increased microbial contamination.	Not mentioned in the study
To evaluate the efficacy of autologous PRP gel in DFU treatment [30].	Randomized control trial. 56 patients with clean chronic DFUs.	Au-RPR gel is more effective than local antiseptic for healing rate and decreasing infection.	Not mentioned in the study
To evaluate the efficacy of au-PRP in DFU healing [56].	Randomized control trial. 90 patients with clean DFUs (47 control and 43 PRP group).	PRP significantly increased the healing rate of DFUs regardless of the age, gender, or smoking and blood pressure status of patients.	Not mentioned in the study
To evaluate the role of PRP in DFU healing [57].	Prospective study. 55 DFU patients (29 PRP and 26 control group).	PRP shortened the time of healing and wound score was significantly improved compared to debridement and dressing only.	Not mentioned in the study
To study the efficacy of au-PRP in managing chronic DFUs [58].	Single-center research. 72 patients with chronic DFUs (activated PRP injection and gel vs. conventional dressing).	Significantly improved healing rate and shorter healing duration in the PRP group.	Not mentioned in the study
To assess the role of au-PRP in promoting non-ischemic DFU healing [59].	Prospective randomized controlled study. 80 patients (40 PRP injections and 40 moist dressings with or without collagenase ointment).	No amputation in the PRP group while 4% amputation in the control group. PRP accelerates wound healing and decreases wound infection.	Not mentioned in the study
To evaluate the role of autologous PRP gel in DFU treatment [60].	Randomized control trial. 24 DFU patients.	Only 25% of patients in the PRP group (3/12) achieved complete healing, while none in the saline group did.	Not mentioned in the study
To evaluate the efficacy of autologous platelet-rich gel in the treatment of deep sinus tract wounds from diabetic ulcers [61].	Clinical case-control study. 48 patients with DFUs (25 in plasma gel and 23 in the conventional dressing group).	No significant differences in the wound conditions between the two group; however, plasma gel can accelerate deep sinus tract healing.	Not mentioned in the study
To examine the safety and effectiveness of topical autologous platelet-rich gel (APG) in DFU healing [62].	Prospective, randomized controlled trial. Total diabetic ulcers (n = 117) and subgroup diabetic foot ulcers (n = 103).	Topical APG + standard treatment is safe and quite effective on chronic refractory DFUs compared with standard treatment.	Not mentioned in the study
To examine the effectiveness of injected au-PRP in DFU healing compared to conventional dressing [63].	Prospective observational study. 80 patients each in PRP and control group.	PRP is significantly effective in wound healing. Wound healing in females and patients with age >55 years is better while using PRP injection.	Not mentioned in the study
To compare the efficacy of au-PRP compared to conventional dressing (normal saline/povidone-iodine)in DFU [64].	Clinical trial. 174 DFU patients (87 in each group).	PRP enhances wound healing, decreases healing time, and makes wounds viable for treatment when wounds are chronic.	Not mentioned in the study
To assess the efficacy of au-PRP and compare the effect of topical and perilesional injections of PRP [65].	Single-center, prospective, interventional study. 60 patients divided into 2 groups.	Perilesional injection is better than topical PRP application in enhancing wound healing of DFU.	Not mentioned in the study

## Data Availability

Not applicable.
